# Investigating the Efficacy of Various Handwashing Methods against Enveloped and Non-Enveloped Viruses

**DOI:** 10.4269/ajtmh.22-0287

**Published:** 2023-02-13

**Authors:** Claire E. Anderson, Jingyan Tong, Winnie Zambrana, Alexandria B. Boehm, Marlene K. Wolfe

**Affiliations:** ^1^ Department of Civil and Environmental Engineering, Stanford University, Stanford, California;; ^2^ Gangarosa Department of Environmental Health, Rollins School of Public Health, Emory University, Atlanta, California

## Abstract

Respiratory and diarrheal diseases are leading causes of death worldwide. Handwashing may reduce disease; however, recommended methods (soap and water for 20 seconds) are not always possible, particularly in low-resource settings. The aim of this study is to evaluate handwashing when recommended methods are not feasible, including washing with water only, washing with soapy water, washing for a short duration, using alcohol-based hand sanitizer (ABHS), and cleaning hands with towels. To evaluate laboratory efficacy, we seeded MS2 (a non-enveloped virus) and Phi6 (an enveloped virus) onto the hands of volunteers who then washed their hands. Viruses remaining were recovered and quantified using culture-based and molecular methods to determine the log reduction value (LRV) after washing. Results indicated that washing with water only and with soapy water were similar to washing with soap and water for 20 seconds for both viruses (median LRV for MS2 = 2.8; Phi6 = 3.2). Most towel alternative conditions had LRVs significantly smaller than LRVs from washing with soap and water for either virus. LRVs of ABHS and soap and water for 5 seconds were similar to soap and water for 20 seconds for Phi6 but less for MS2 (median MS2 LRV differences = 2.5 and 0.51 for ABHS and soap and water for 5 seconds, respectively). Additionally, LRVs determined using molecular methods were in agreement with those obtained using culture-based methods. These results suggest some handwashing alternatives were as effective as recommended methods whereas others were not, and inform recommendations and future research on handwashing alternatives in low-resource settings.

## INTRODUCTION

In 2019 the World Health Organization (WHO) reported that respiratory infections and diarrheal diseases are two of the top 10 leading causes of death worldwide.^1^ Diarrhea and acute lower respiratory tract infections are of particular concern in low- and middle-income communities and for children less than 5 years, with 3.5 million dying each year from these diseases.^2^^,^^3^ Respiratory infections and diarrheal diseases are caused by several pathogen types, including bacteria like enterotoxigenic *Escherichia coli* (ETEC), protozoa like *Giardia *spp., non-enveloped viruses like norovirus, and enveloped viruses like SARS-CoV-2. Pathogens can be transmitted through direct transmission (direct contact or droplet spread) or may spread through indirect transmission (contaminated food, water, or inanimate surfaces).^3^^–^^6^ Hands play a role in indirect transmission; they can act as vehicles for pathogens between infected individuals and as environmental reservoirs to susceptible individuals.^6^^,^^7^

Hand hygiene has been suggested as the most cost-effective and efficient means of reducing the global burden of disease by the WHO and the Centers for Disease Control and Prevention (CDC).^8^^,^^9^ The WHO recommends washing hands with soap and water for at least 40 seconds, whereas the CDC recommends 20 seconds.^10^^,^^11^ In a laboratory setting, handwashing efficacy studies using soap and water have consistently shown a > 2 log reduction value (LRV) in organisms after handwashing.^12^^–^^17^ Hand hygiene has also been shown to reduce disease: promoting hand hygiene was found to reduce respiratory illness by 21–24%^7^^,^^18^ and enteric disease by 31–40%.^8^^,^^18^

Despite this, a 2014 study found that only 19% of people worldwide are estimated to wash their hands with soap and water after contact with excreta.^8^ Prevalence of handwashing is higher in high-income countries (48–72%) than in low- and middle-income regions (5–25%),^8^ possibly due to a lack of access to handwashing supplies in the low- and middle-income countries. This is also a challenge among people experiencing homelessness, among refugees and internally displaced people, and in remote areas. In 2019, approximately 26% of the global population (1.6 billion people) lacked access to soap and water for handwashing.^4^^,^^19^

When handwashing with soap and water for a minimum of 20 seconds is not realistic, alternative handwashing techniques must be considered. A common alternative handwashing method is alcohol-based hand sanitizer (ABHS). In instances when handwashing with soap and water is not available, both the WHO and CDC recommend hand sanitizer^10^^,^^11^; however, ABHS can be costly to buy and transport, must be readily refilled, and may not be consistently available in low-resource communities.^16^^,^^20^^,^^21^ Additionally, ABHS has limited efficacy on soiled hands and is not effective for all viruses.^4^^,^^5^^,^^17^^,^^18^^,^^22^ Other alternatives are not widely recommended for handwashing but are often practiced and may be preferable to not washing at all, including washing with water alone, washing with ash or sand, or using an alternative durable and reusable consumer product. One such product is the Supertowel (Real Relief, Kolding, Denmark), a microfiber towel with an antimicrobial fabric treatment. The Supertowel reportedly cleans the user’s hands, inactivates pathogens, and can be reused for months at a time.^23^

In order for a handwashing alternative to be promoted, it should perform as well as recommended methods at removing pathogens. However, there is little data on the performance of alternative handwashing methods, particularly for enveloped viruses. Although other studies have considered the effectiveness of alternative handwashing techniques with bacteria, protozoa, and non-enveloped viruses,^4^^,^^5^^,^^17^^,^^18^^,^^22^^,^^24^ these mostly focus on ABHS. To date we only identified four studies examining the removal of enveloped viruses from hands during handwashing, and among these the only alternatives outside soap and water used were alcohol-based disinfectants and water only.^12^^–^^16^

Several methods can be used to measure the effectiveness of handwashing methods, including the use of culture-based^12^^–^^16^^,^^24^^–^^26^ or molecular methods for quantification^5^^,^^26^^,^^27^ of pathogens or pathogen indicators on seeded^5^^,^^12^^–^^16^^,^^25^^,^^26^ or unseeded hands.^2^^,^^4^^,^^18^^,^^24^^,^^27^ Many human pathogenic viruses are difficult to culture, requiring specialized space and equipment, and as a result molecular methods like polymerase chain reaction (PCR) may be easier to use. Molecular methods, however, are unable to directly measure infective viruses, and the relationship between the concentration of virus measured by molecular methods and the concentration of infective virus measured through culture-based techniques is generally unknown.

The goal of the present study is to investigate the efficacy of different handwashing methods against both non-enveloped and enveloped viruses using both culture-based methods and molecular methods. In addition to testing washing with soap and water for 20 seconds, we tested alternatives including using ABHS, washing with soapy water, washing for a shorter amount of time (5 seconds), washing with water only, washing with an untreated microfiber towel, and washing with the Supertowel. To evaluate efficacy of the selected methods, handwashing studies were carried out with volunteers using surrogate biosafety level 1 viruses MS2 (non-enveloped) and Phi6 (enveloped).

## MATERIALS AND METHODS

### Volunteers.

Twenty-six volunteers consented and participated in the study. Volunteers were allowed to participate if they self-reported as healthy, were between the ages of 18 and 65, and had no visible sores on their hands or fingerpads. The age, sex, hand length, and hand breadth of the volunteers were recorded. Hand length and breadth were recorded according to procedures of the National Aeronautics and Space Administration.^28^ Volunteers were asked to remove rings, watches, and bracelets prior to the start of experiments. Volunteers were enrolled with approval from the Stanford University Research Compliance Office for Human Subjects Research (IRB-58905). Given the sample size (26 volunteers compared for plaque assays and 5 volunteers compared for reverse transcription–quantitative PCR [RT-qPCR]), a specificity of 95%, and a power of 80%, the study is powered for a Cohen’s d effect size of 0.41 for plaque assays and 1.03 for RT-qPCR. Cohen’s d effect size is a unitless value used to describe the standardized mean difference of an effect. Typically, 0.2, 0.5, and 0.8 are delimiters of small, medium, and large effect sizes, respectively.^29^^,^^30^ Therefore, our study was powered for small effect sizes between handwashing methods for plaque assays and large effect sizes between handwashing methods for RT-qPCR.^16^^,^^25^

### Handwashing methods.

The 12 handwashing methods tested consisted of one handwashing test with 1) ABHS, four handwashing tests with a combination of soap and water: 2) soap and water for 20 seconds, 3) soap and water for 5 seconds, 4) soapy water for 20 seconds, 5) water only for 20 seconds, one test with 6) a regular microfiber towel, and six Supertowel tests: 7) new dry Supertowel, 8) new wet Supertowel, 9) new dirty Supertowel, 10) used dry Supertowel, 11) used wet Supertowel, and 12) used dirty Supertowel. A no-wash control was included for each volunteer and consisted of application of the virus followed by recovery.

### Virus preparation.

Phi6 (NBRC 105899) and its host *Pseudomonas syringae* (ATCC 21781) were kindly provided by K. Wigginton at the University of Michigan. To propagate *P. syringae*, 30 mL of nutrient broth was inoculated with 20 μL of *P. syringae* stock and incubated at 30°C while shaking at 75 rpm for 48 hours until use. Phi6 virus stock was created using the method described in Anderson and Boehm.^6^ Briefly, soft agar from a lysed plaque assay plate was added to phosphate-buffered saline (PBS; Fisher BioReagents). The agar–PBS mixture was then filtered (0.2-μm pore size), concentrated using an Amicon^®^ Ultra-15 centrifugal filter unit (EMD Millipore), and stored at –80°C. Phi6 stock concentration was approximately 10^11^ plaque-forming units (PFU)/mL.

MS2 (DMS No. 13767) was propagated in its host *E. coli* (ATCC 700891). Twenty milliliters of tryptic soy broth (TSB; pH 7.3 ± 0.2) was inoculated with 20 μL *E. coli* stock, incubated (without shaking) at 37°C until the growth phase was logarithmic, and used immediately for experiments. Logarithmic growth phase, as measured by a spectrophotometer at a wavelength of 600 nm, was reached about 3 hours after inoculation. MS2 virus stock was created from the lysed plaque assay plate soft agar–PBS mixture, which was filtered (0.2-μm pore size) and stored at −80°C. Ultrafiltration was not used to concentrate MS2 because the concentration without it was adequately high. MS2 concentration of the stock was approximately 10^10^–10^11^ PFU/mL.

### Experimental procedure.

**Figure 1.**
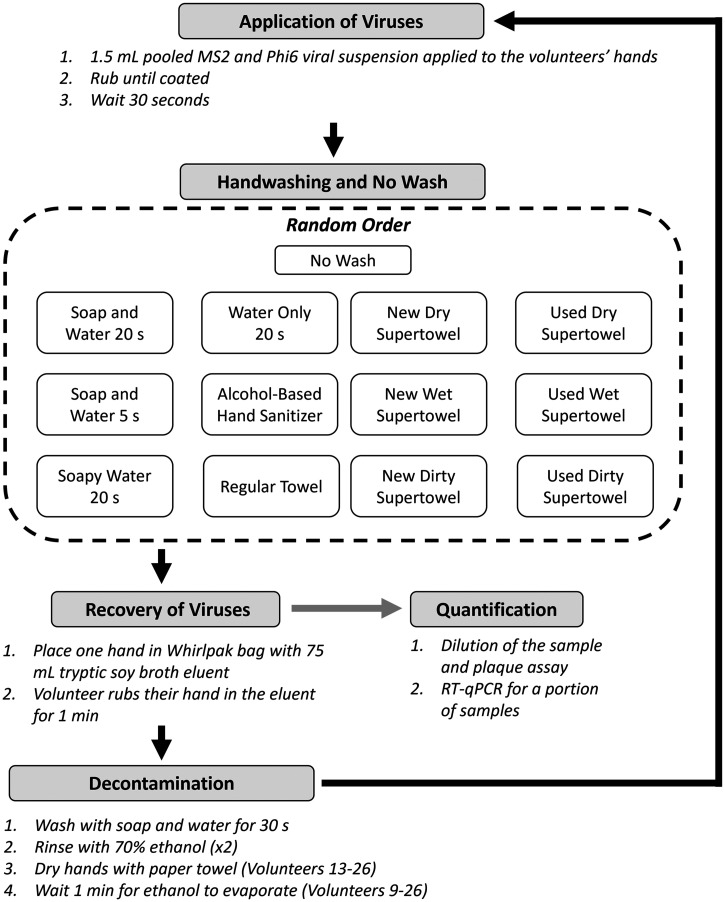
Outline for volunteer experiments. Experiments begin with decontamination, then move to application of the virus. After virus application, a handwashing method is implemented, and the virus is recovered. The process is then repeated for all handwashing methods. After all methods are performed, the virus is quantified.

The experimental procedure for the volunteer handwashing experiments is based on Wolfe et al.^16^ Twelve different handwashing methods and a no-wash control were applied to the volunteer in a random order. Each handwashing method consisted of application of virus mixture, handwashing, TSB recovery from the hands of the volunteer, and decontamination (Figure 1).

#### Initial decontamination and virus application.

Temperature and humidity of the laboratory were recorded prior to each volunteer experiment using a ThermoPro TP49 digital hygrometer. Volunteers were asked to wash their hands at the start of experiments for 30 seconds using liquid soap (Colgate-Palmolive Company, New York, NY) and water and to dry their hands with a paper towel (Kimberly-Clark, Irving, TX). After washing and drying, 1.5 mL of pooled Phi6 and MS2 virus stock (750 μL of approximately 10^9^ PFU/mL Phi6 combined with 750 μL of approximately 10^9^ PFU/mL MS2) was applied to the volunteers’ hands. The volunteers rubbed their hands together until the virus solution coated the front and backs of their hands.

#### Handwashing.

Handwashing for each condition was conducted as follows. Approximately 1.5 mL of ABHS (62% ethyl alcohol; Target Corp., Minneapolis, MN) was used per volunteer. Handwashing with soap and water also used approximately 1.5 mL of liquid soap (Colgate-Palmolive Company). Soapy water was created by adding 30 g of powdered laundry detergent (Procter & Gamble, Cincinnati, OH) to 1.5 L of tap water. Soapy water flowed from a tank tap at approximately 0.08 L/s; flow from the sink tap had a flow of approximately 0.09 L/s. The Supertowel was a 30-cm^2^ microfiber towel that, according to the manufacturer, had been treated with a permanently bonded antimicrobial layer. A used Supertowel was one that was washed by hand and air dried 10 times (see supplemental materials for details). A wet Supertowel was completely submerged in tap water and then rung out to leave the towel damp. A dirty towel was wetted with dirty water made with 5 g of autoclaved soil (Gro Tec, Inc., Madison, GA) suspended in 1 L of tap water and then rung out to leave the towel damp. A regular towel was a 30-cm^2^ microfiber towel without antimicrobial treatment (Real Relief). Additional details are provided in the supplemental materials.

#### Recovery.

After handwashing, volunteers placed one of their hands (randomly chosen through a random number generator and kept consistent for all handwashing methods) into a 55-oz. Whirl-Pak bag (Whirl-Pak, Madison, WI) containing 75 mL of autoclaved TSB. The volunteers then moved their hand gently back and forth in the bag for 1 minute. Viral removal is assumed to be equal on both hands.

#### Decontamination.

To decontaminate hands in preparation for the next test, volunteers washed their hands with soap and water for 30 seconds and applied two rounds of 70% (v/v) ethanol (Thermo Fisher Scientific, Waltham, MA). After the volunteers reported that the ethanol had evaporated, ethanol was applied a second time to the volunteers’ hands. After the second ethanol wash dried, volunteers 1–8 proceeded directly to the application of virus for the next handwashing method. Volunteers 13–26 used a paper towel to dry their hands after the second ethanol application, and volunteers 9–26 waited 1 minute after the second ethanol application before proceeding to the application of virus step. We added the additional wait time for volunteer 9 because, in a few instances, viruses were not recovered in the no-wash control during the work with volunteers 1–8, and we wanted to ensure that this was not being caused by residual ethanol left on the hands. Changes ensured ethanol was removed or evaporated prior to application of the virus. Retrospective analysis of the no-wash control results suggested that this change had no effect on virus recovery from the no-wash control (see supplemental materials). Data from all volunteers were retained for subsequent analyses. Viral contamination from the environment was deemed unlikely, and testing of decontamination procedures indicated they were effective (details in supplemental materials).

#### Sample analysis.

Of the 75 mL of TSB collected, 1 mL was used for plaque assays. An additional three 1-mL aliquots were reserved for RT-qPCR and stored at −80°C for 1–6 months until RNA extraction. Samples were thawed once prior to RNA extraction. RT-qPCR was performed on samples from the last five volunteers for the following handwashing methods: new wet Supertowel, new dry Supertowel, soap and water for 20 seconds, water only for 20 seconds, and no-wash control.

### Virus plaque assay quantification.

Phi6 and MS2 were enumerated in samples using plaque assays following Anderson and Boehm.^6^ Briefly, a double-layer agar technique was used for both. For Phi6, 100 μL of *P. syringae* and 100 μL of sample were added to soft nutrient agar (0.3% agar) and then poured onto hard nutrient agar plates (2.3% agar). For MS2, 200 μL of *E. coli* and 300 μL of sample were added to soft tryptic soy agar (0.7% agar) and then poured onto hard tryptic soy agar plates (1.5% agar). Phi6 and MS2 plates were incubated at 30 and 37°C, respectively, overnight before plaque forming units (PFUs) were counted.

We assayed sample dilutions ranging from undiluted to 10^−4^ for Phi6 and undiluted to 10^−5^ for MS2. Samples were not run with technical replicates but were assayed with multiple dilutions chosen based on pilot experiments. The countable number of PFUs ranged from 1 to 500; we were able to count up to 500 PFU due to the small size of plaques. If multiple dilutions had countable plaques, data from those dilutions were used to calculate LRVs (see data analysis section below). Plates with > 500 PFUs were classified as too numerous to count (TNTC). If all dilutions assayed yielded > 500 PFUs, greater dilutions were run on the following day using samples archived at 4°C to achieve countable plaques. In instances where no PFU were detected in the undiluted and diluted samples, 0.5 (half the limit of detection, 1 PFU) was substituted as the undiluted sample PFU. All samples were initially assayed within 24 hours of sample collection.

Positive and negative controls were run for each plaque assay on each day samples were analyzed and are described in the supplemental materials.

### Virus RT-qPCR assay quantification.

RNA extraction prior to RT-qPCR was completed with the QIAamp Viral RNA Mini kit (Qiagen) using 140 μL of sample and yielding 80 μL of viral RNA. Molecular-grade water was used as RNA extraction–negative controls. MS2 and Phi6 stocks were used as RNA extraction–positive controls. RNA extractions occurred in five sets. RNA extraction–negative and RNA extraction–positive controls were tested at the start of RT-qPCR experiments (set 1) and at the conclusion of RT-qPCR experiments (set 5). Inhibition tests for a subset of samples revealed samples were not inhibited at a 1:10 dilution (see supplemental materials); all samples were diluted 1:10 prior to RT-qPCR.

RT-qPCR was performed for one genome target for MS2 and one target for Phi6, with targets described in Loeb et al.^31^ and Ye et al.^32^ (Table 1), respectively, using a StepOnePlus™ real-time PCR system. Target size and base pair composition is described in the supplemental materials. RT-qPCR was performed using the Gotaq OneStep RT-qPCR kit (Promega). Reaction setup for MS2 consisted of 10 μL GoTaq qPCR Master Mix, 0.4 μL GoScript RT Mix for one-step RT-qPCR, 0.17 μL bovine serum albumin (BSA), 0.33 μL CXR reference dye, 3.9 μL nuclease-free water, 1.2 μL primer pool (10 μM forward and reverse primer), and 4 μL or RNA extract to yield a final reaction volume of 20 μL. The reaction setup for Phi6 included equal amounts of GoTaq qPCR Master Mix, GoScript RT Mix for one-step RT-qPCR, CXR reference dye, primer pool, and RNA extract as MS2. Phi6 differed in that 4 μL betaine was used instead of BSA and nuclease-free water was adjusted to 0.07 μL to yield 20 μL per reaction.

**Table 1:** Genome target sequences for Phi6 and MS2. Forward and reverse sequences are shown for the MS2 target (MP6) and Phi6 target (M)

Genome target name	Forward sequence (5′–3′)	Reverse sequence (5′–3′)
MP6	CCTAAAGTGGCAACCCAGAC	AAAGATCGCGAGGAAGATCA
M	CCTGAGGAAACGGCTCAACT	CATAGCCAACGAACTGCTGC

Thermocycling conditions for MS2 were as follows: reverse-transcriptase step at 40°C for 15 minutes, a PCR activation step at 95°C for 10 minutes, followed by 40 cycles of denaturing (95°C for 10 seconds), annealing (59°C for 30 seconds), and extension (72°C for 30 seconds). Thermocycling conditions for Phi6 included an initial denaturing step (99°C for 5 minutes) prior to reverse transcriptase. Next, reverse transcriptase proceeded at 40°C for 15 minutes, followed by a PCR activation step at 95°C for 10 minutes, 40 cycles of denaturing (95°C for 15 seconds), annealing (59°C for 30 seconds), and extension (72°C for 40 seconds). After cycling both Phi6 and MS2, melt curves were developed by increasing the temperature from 60 to 95°C for 10 minutes.

For Phi6 and MS2, cDNA standards were prepared by extracting RNA from an unthawed handwashing TSB sample, amplifying each target through RT-qPCR, visualizing the product using gel electrophoresis, extracting the cDNA from the gel, and diluting the cDNA appropriately (details in the supplemental materials). Standard curve efficiency and *R*^2^ values for each target were: MP6 86% (SD = 0.02), 0.999 (SD = 0.001); M 58% (SD = 0.03), 0.99 (SD = 0.002).

RT-qPCR samples and standards were run in duplicate with no-template controls for each plate. No-template controls and negative extraction controls were required to have no contamination. Duplicate samples were required to be within a half of a standard deviation cycle threshold (Ct) from one another; if they failed, samples were rerun. Ct thresholds were set at 0.5 for Phi6 and 1 for MS2. Automatic baselines were used on the RT-qPCR machine prior to data exportation.

### Data analysis.

Log reduction values in the quantity of viruses on hands was the main outcome of these experiments:

$$\text{LRV}=-{\text{log}}_{10}\left(\frac{N}{N_{0}}\right)$$ (1)

where *N* was the amount of virus measured on hands after a handwashing treatment, and *N*_0_ was the amount of virus recovered when no handwashing treatment is applied. LRVs were calculated for both methods of quantifying the amount of virus using culturing methods to quantify the number of PFUs and using molecular methods to quantify the viral gene copies. In Equation 1, we assumed that the recovery of virus from hands, defined as the fraction of virus on hands recovered using the TSB recovery method, was the same after handwashing methods and after the no-wash control and therefore had no impact on the LRV. LRV was calculated for each volunteer for each handwashing method.

*N*/*N*_0_ was calculated as follows using the plaque assay results:

$$\frac{N}{N_{0}}=\frac{\sum^{\text{​}}\;\left({\text{PFU}}_{\text{H}}*D_{\text{H}}\right)}{\sum^{\text{​}}\left({\text{PFU}}_{\text{NW}}*D_{\text{NW}}\right)}*\frac{n_{\text{NW}}}{n_{\text{H}}}$$ (2)

where dilutions with countable PFU for each handwashing method (PFU_H_) were multiplied by their appropriate dilutions (*D*_H_) and summed. The results were divided by the sum of the product of the PFU from the no-wash control (PFU_NW_) and appropriate dilutions (*D*_NW_) over all countable dilutions. The ratio of the number of countable dilutions from the no-wash control and handwashing method (*n*_NW_/*n*_H_) is then multiplied to obtain the fraction of virus remaining. For RT-qPCR, *N*/*N*_0_ was calculated for both viral targets; *N* was equal to the average concentration (copies/reaction) of the technical duplicates for each handwashing method, and *N*_0_ was the average concentration (copies/reaction) of the technical duplicates from the no-wash control.

Statistical analysis was performed with R (version 1.2.5042; R Foundation for Statistical Computing, Vienna, Austria). Normality was assessed, and plaque assay as well as RT-qPCR LRV data were not normally distributed (see supplemental materials for details). A Friedman test was used to determine if there were any significant differences in LRVs among the 12 handwashing methods. To determine which methods had LRVs that were significantly different from one another, a post hoc Wilcoxon signed-rank test was performed. Separate tests were run for each of the four endpoints (MS2 and Phi6 for culture and RT-qPCR results). Comparisons between endpoints were made with Wilcoxon signed-rank tests. A Bonferroni correction was implemented in the Wilcoxon signed-rank tests to adjust for multiple comparisons, and corrected *P* values are reported. *P* values < 0.05 are considered significant (α = 0.05). Wilcoxon signed-rank test effect sizes were calculated as the sample z-score divided by the square root of the sample size.

## RESULTS

### General.

Twenty-six volunteers participated in the handwashing experiments; 14 and 12 self-identified as cisgender-female and cisgender-male, respectively. Their age ranged from 19 to 56 years (median = 26 years); hand length ranged from 15.3 to 22.5 cm (median = 18.5 cm), and hand breadth ranged from 6.4 to 10.8 cm (median = 8.3 cm). Room temperature during the experiments ranged from 20.1 to 21.4°C (median = 21.0°C), and relative humidity ranged from 39 to 68% (median = 59%). Complete temperature and humidity data, as well as the hand chosen for recovery and order of handwashing methods for each volunteer, are in the supplemental materials.

All plaque assay positive controls had concentrations similar to MS2 and Phi6 stock concentrations (approximately 10^11^ PFU/mL), and all negative controls were negative. There were no instances where all dilutions were classified as TNTC. Of 338 undiluted samples for Phi6, there were 135 instances in which 0 PFU were detected, and therefore 0.5 was substituted as the undiluted sample PFU. There were no instances in which 0 PFU were detected for MS2 in the undiluted sample. All RNA extraction–negative controls and RT-qPCR no-template controls had no contamination. RNA extraction–positive controls had high concentrations (approximately 10^12^ copies/mL of viral TSB stock), similar to MS2 and Phi6 plaque assay stock concentrations (PFU/mL). Additional information regarding the Environmental Microbiology Minimum Information Guidelines for qPCR^33^ is provided in the supplemental materials.

### Plaque assay.

**Figure 2.**
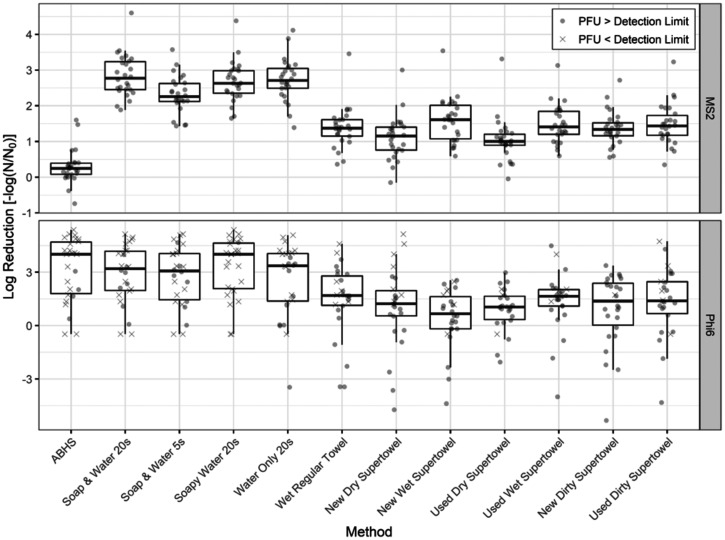
Box plot of plaque assay log reduction values for each handwashing method, overlayed with jittered data points from individual experiments. The box is made up of the 25th quartile, median, and 75th quartile. The length of each whisker is 1.5 times the interquartile range (IQR). Values outside the IQR criterion (the first quartile minus 1.5 times the IQR to the third quartile plus 1.5 times the IQR) are considered outliers. Circles are data points where plaque-forming units (PFU) that were above the limit of detection (1 PFU for the undiluted sample) for the handwashing method (from Equation 2). Crosses are data points where PFU from the handwashing method were below the limit of detection and were replaced with 0.5 PFU. ABHS = alcohol-based hand sanitizer.

The distributions of LRVs for each handwashing method and virus (LRV median, quartiles, and outliers) are provided in Figure 2. A total of 624 LRV data points were plotted (26 volunteers × 12 handwashing methods × 2 viruses). The median LRV across all handwashing methods for MS2 was 1.5. The median LRV across all handwashing methods for Phi6 was 1.7. The 12 Wilcoxon signed-rank tests (with a Bonferroni correction) performed to assess the difference between results from the two viruses for each handwashing method showed that the LRVs were significantly different between MS2 and Phi6 for ABHS (*P* = 2.1 × 10^−6^) and the new wet Supertowel (*P* = 9.4 × 10^−4^). The median ABHS LRV was 3.8 more for Phi6 than for MS2, and the median new wet Supertowel LRV was 0.94 less for Phi6 than MS2. All other handwashing method LRVs were not significantly different from one another between MS2 and Phi6 (*P* > 0.08).

Negative LRVs were observed for some conditions, indicating that the number of PFU recovered from the hand after the handwashing method was greater than the number of PFU recovered from the hand for the no-wash control. We retained these values because they captured variability of the experimental procedure and controls performed as expected.

For each virus, there were statistical differences in LRVs between handwashing methods (Friedman test, *P* < 10^−10^ for both MS2 and Phi6) (Figure 2). All effect sizes and significant *P* values from statistical tests between handwashing methods are shown in full in a heat map in the supplemental materials. For MS2, all towel handwashing methods had significantly lower LRVs than all water and/or soap methods for MS2. The median difference in LRVs between water and/or soap methods and towel methods for MS2 was 1.3 (*P* < 6.9 × 10^−4^); for Phi6, the median difference was 2.0 (*P* < 3.0 × 10^−3^). Among the towel methods for MS2, the new dry Supertowel and the used dry Supertowel had lower LRVs than a majority of the other towel methods (*P* < 0.04). The median difference between the new dry Supertowels and all other towel differences was 0.19; the median difference between the used dry Supertowels and all other towel differences was 0.38. For Phi6, all Supertowel conditions had lower LRVs than conditions with soap and water (washing with soap and water for 20 seconds, washing with soap and water for 5 seconds, and washing with soapy water). A subset of Supertowel for Phi6 conditions was not significantly different from washing with water alone. For Phi6, washing with a regular towel LRVs were lower than washing with soapy water (*P* = 0.01) and washing with soap and water for 5 seconds (*P* = 0.02) but were similar to washing with soap and water for 20 seconds (*P* = 0.06) and washing with water alone (*P* = 0.25). Overall, all towel method LRVs were similar to each other for Phi6 (*P* > 0.07).

Among methods with soap and water, washing with soap and water for 5 seconds had a lower LRV than washing with soap and water for 20 seconds or soapy water for MS2; the median difference in LRVs between soap and water for 5 seconds and the other soap methods was 0.44 (*P* < 0.01). The remaining soap and/or water methods (washing with soapy water and washing with water only) were not significantly different from washing with soap and water for 20 seconds for MS2 (*P* = 1.0 for both post hoc tests). There was no significant difference in LRVs between water and/or soap methods for Phi6, including the use of water only (*P* > 0.18).

Finally, ABHS had similar LRVs to water and/or soap methods for Phi6 (post hoc test, all *P* > 0.25) but had significantly lower LRVs than water and/or soap methods for MS2 (post hoc test; *P* = 2.3 × 10^−6^ for all six tests). For MS2, the median difference between water and/or soap methods and ABHS was 2.4. ABHS was also associated with significantly lower LRVs than towel methods for MS2 (post hoc test; *P* < 1.3 × 10^−4^). For MS2, the median difference between towel methods and ABHS was 1.1.

### LRVs as measured by RT-qPCR.

**Figure 3.**
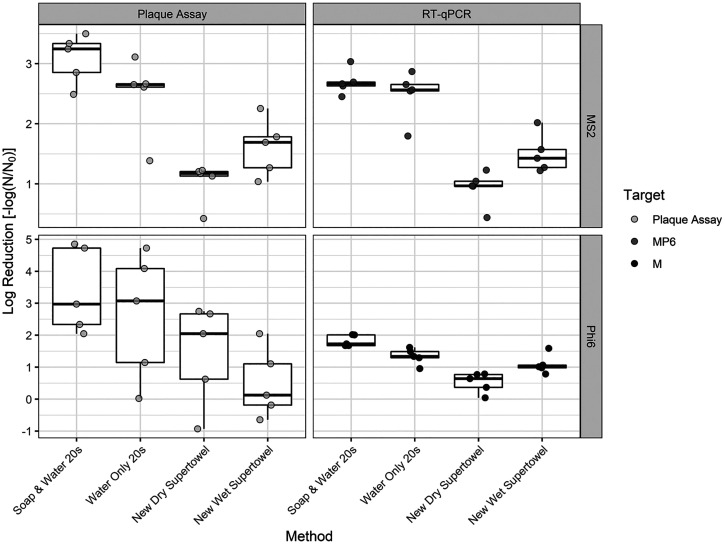
Box plot of log reduction values from the five volunteers tested for both plaque assays and reverse transcription–quantitative polymerase chain reaction (RT-qPCR). Overlaid on the box plots are jittered data points. The box is made up of the 25th quartile, median, and 75th quartile. The length of each whisker is 1.5 times the interquartile range (IQR). Values outside the IQR criterion (the first quartile minus 1.5 times the IQR to the third quartile plus 1.5 times the IQR) are considered outliers. The axes for MS2 and Phi6 differ.

We used RT-qPCR to quantify RNA genome fragments from MS2 and Phi6 in a subset of the handwashing methods from volunteers 21, 22, 24, 25, and 26, and subsequently we calculated LRVs for the different genome fragments (Figure 3). Overall, median LRVs were between 2.66 and 0.64, depending on handwashing method and viral target. The LRVs obtained using RT-qPCR and plaque assays for each handwashing method from the same set of volunteers (volunteers 21, 22, 24, 25, and 26) are plotted in Figure 3. Results from RT-qPCR were compared with plaque assay results by using Wilcoxon signed-rank tests. LRVs for each handwashing method were not different as measured by plaque assay and RT-qPCR for either virus (*P* > 0.06 for Phi6; *P* > 0.13 for MS2). Wilcoxon effect sizes between RT-qPCR and plaque assays for each handwashing condition were >0.54 (medium to large effects) for all handwashing methods except for water only for 20 seconds for MS2 (effect size = 0.18, small effect).

## DISCUSSION

We investigated the efficacy of handwashing with a focus on critical research gaps: 1) removal of viruses during handwashing and 2) handwashing methods that are realistic and commonly used in low-resource and crisis settings yet in some cases fall outside internationally recommended standards. We found that several alternative handwashing methods, but not all, were as effective in a laboratory setting for removal and inactivation of viral surrogates as washing with soap and water for 20 seconds. For both MS2 and Phi6, use of soapy water and water alone for 20 seconds were similar to washing with soap and water for 20 seconds. This is in agreement with a previous study that found that washing with soapy water was as effective as bar soap for thermotolerant coliforms^24^ and supports WHO recommendations that soapy water is an approved alternative to soap and water for handwashing in the COVID-19 pandemic.^4^ Soapy water costs less, is more easily stored, uses less water, and is less easily stolen than liquid or bar soap.^24^ Previous studies also found that washing with water only was effective for norovirus^5^ but may not be effective for some fecal indicators, like thermotolerant coliforms.^24^ Reducing washing time to 5 seconds did not change LRVs for Phi6, similar to findings showing that increasing the time of washing from 15 to 30 seconds did not improve removal of coliforms.^24^ However, reduced wash time did result in a lower LRV for MS2, indicating that reduced washing time may not be appropriate for all pathogens.

Alcohol-based hand sanitizer is commonly recommended as an alternative to soap and water; we observed that it was effective for enveloped viruses but not for non-enveloped viruses. This was expected based on previous work showing that ABHS is effective in laboratory settings at reducing the concentration of enveloped viruses, including influenza,^12^^,^^14^^–^^17^ and work showing that ABHS is less effective for non-enveloped viruses like norovirus and has limited efficacy on soiled hands.^4^^,^^5^^,^^17^^,^^18^^,^^22^ Reducing infectivity of non-enveloped viruses is of particular concern because norovirus and rotavirus, two non-enveloped viruses, account for 26% of all deaths due to diarrheal disease^3^; in 2012, inadequate hand hygiene was estimated to result in almost 300,000 diarrheal deaths.^34^ Results from this study support the conclusion that using ABHS for handwashing during a non-enveloped viral disease outbreak may not be effective at interrupting transmission.

Overall, Supertowel methods were less effective for handwashing than methods using soap and water, with the exception that some Supertowel variations were not significantly different from use of water only for the enveloped virus. All Supertowel variations were as effective or less effective than a towel without treatment of MS2 and Phi6. Washing with a regular towel was similar to washing with soap and water for 20 seconds and water alone for Phi6; however, overall, results suggest that handwashing using towel methods are unlikely effective at rmoving viral pathogens. A previous study found that the Supertowel had similar LRVs to washing with soap and water for bacteria,^25^ but results for viruses appear to differ, and results reported here align with a 2021 study using SARS-CoV-2, Phi6, MHV, and MS2 found that the Supertowel was not an effective antimicrobial surface.^35^

Log reduction values calculated using RT-qPCR results generally reflected those calculated using plaque assay results. Although plaque assays are useful to quantify infectious viruses, they are often infeasible for human pathogenic viruses due to host cell culturing difficulties and the risk of infection to those working in the laboratory. As a result, RT-qPCR methods are commonly used to quantify health-relevant targets of interest, despite the fact that their relation to the presence of infectious viruses is generally unknown. Our results suggest that RT-qPCR LRV data may agree with results from plaque assays, supporting the use of RT-qPCR to estimate removal of viruses from hands during handwashing. This study was powered for large effect sizes for RT-qPCR results. Although most of the RT-qPCR effect sizes measured in this study were medium to large effects and we were able to determine there was no significant difference between RT-qPCR and plaque assays, future studies may consider increasing sample size in the RT-qPCR and plaque assay comparison to power the study for small to medium effect sizes.

There were some technical limitations to this study. In addition to the need to use bacteriophages as surrogates to protect the health of participants, there were some other factors that might affect estimated LRVs. First, because 135 of 338 Phi6 samples were non-detects and were replaced with 0.5 PFU, the LRVs we found are likely underestimates of the true LRVs that some handwashing conditions were able to achieve. Particularly for ABHS and the soap and water conditions, where a majority of samples were non-detects for Phi6, the true LRV may be higher. Future studies may consider concentrating the handwashing recovery eluent or increasing the viral concentration applied to the hands to be able to measure these high LRVs. Additionally, tap water, which may contain disinfection residuals, was used in the handwashing experiments. Chlorine levels < 1 ppm have been shown to kill bacteria,^36^ and chlorine levels up to 4 ppm are permissible by the USEPA for drinking water.^37^ A previous study found that, at a higher concentration (500 ppm), handwashing with a chlorine solution resulted in enveloped virus LRVs similar to washing with soap and water.^16^ Paper towels used after each handwashing method may have increased observed LRVs because they may physically remove additional virus after washing and before recovery. Paper towels were used after each handwashing method, so we assume the reduction caused by paper towels (if present) was consistent throughout the experiment. Finally, RT-qPCR results were based on testing done immediately after live virus was seeded and recovered; results may vary under other conditions.

Although this study presents alternative handwashing methods, it is important to note that this study evaluates laboratory efficacy and that the effectiveness of a handwashing solution depends on correct and consistent use. There are many ways that user perception, preference, and access may alter the effectiveness and longevity of handwashing interventions,^38^ as demonstrated in the case of one program providing ABHS to a rural community in Bangladesh.^4^ Although ABHS is effective against non-enveloped viruses and participants in a study reported liking it, soap and water were preferred, and cases in which ABHS use was appropriate were not always clear to participants.^4^ ABHS is about three times the cost of bar soap and 30 times the cost of soapy water for a similar number of wash events and was not available in local Bangladesh markets.^4^ Case studies like this are important to keep in mind when placing the results of the study reported here in context.

Laboratory efficacy studies provide important information to inform the recommendation or distribution of handwashing solutions, especially during humanitarian crises, in low- and middle-income countries, and for people experiencing homelessness who may not have access to soap and water and must weigh alternatives. Although laboratory testing results like those available in this study are available for many recommended products against bacteria, these evaluations are not often conducted for alternative handwashing methods or a broad range of types of organisms. Studies like this one provide a valuable foundation for further investigation that should be done to evaluate field performance of methods among vulnerable populations after evaluations show they are effective in a laboratory setting. We recommend that future studies consider additional alternative handwashing methods and continue to use both culture-based methods and molecular methods to further compare the number of infectious viruses to the quantity of genetic material observed for viruses in the environment.

## Supplemental files


Supplemental materials
