# Supplementary material for: Investigating the Efficacy of Various Handwashing Methods against Enveloped and Non-Enveloped Viruses
Source: Am J Trop Med Hyg. 2023 Feb 13;108(4):820–8. doi: 10.4269/ajtmh.22-0287 (PMC10076994; doi:10.4269/ajtmh.22-0287)
Supplement: Supplementary file 1 [file tpmd220287.SD1.pdf]

<u>**Supplemental Materials**</u>

Investigating the efficacy of various handwashing methods against enveloped and non-enveloped viruses

Claire E. Anderson[1], Jingyan Tong[1], Winnie Zambrana[1], Alexandria B. Boehm[1], Marlene K. Wolfe[1,2*]

[1] Department of Civil and Environmental Engineering, Stanford University, Stanford, CA, 94305
[2] Gangarosa Department of Environmental Health, Rollins School of Public Health, Emory University, Atlanta, GA, USA, 30322
*Author to whom correspondence should be addressed: Marlene Wolfe, marlene.wolfe@emory.edu, 617-583-2401

The supplemental material contains the following information, as referenced in the main article:

Supplementary methods………………………………………………………………….. S2
Table S1: Handwashing methods….…………………………………………………. S3
Table S2: Experiment conditions…………..…………………………………………. S5
Figure S1: Experimental setup photos..……………………………………………….. S6
Experimental power calculations………………………………………………………. S7
Table S3: RT-qPCR targets………...…………………………………………….…... S8
Inhibition testing……………………………………………………………………….. S9
  Figure S2: RT-qPCR Inhbition Testing
Environmental Microbiology Minimum Information (EMMI) Checklist………………... S10
MS2 and Phi6 Handwashing Heat Maps……………………………………………... S11
  Figure S3: MS2 Heat Map…………………………………………………... S11
  Figure S3: Phi6 Heat Map……….…………………………………………... S12
Figure S5: Standard curve data…….…….……………………………………………. S13
Distribution of data and normality tests………………………………………………... S14
  Figure S6: MS2 Plaque assay normality tests (all volunteers)…………….……….. S15
  Figure S7: Phi6 Plaque assay normality tests (all volunteers)………….….……….. S16
  Table S4: Normality test results for data subsets……………….……….….…….. S17

Supplementary Methods

Decontamination. The tenth percentile of no wash recovery is equal to 317 PFU/mL for Phi6 and MS2 in aggregate. We report the 10th percentile to represent the portion of data with low recovery from the no-wash condition to compare decontamination methods. A different percentile could have been chosen for this comparison based on author preferences. Before decontamination changes were made (volunteers 1-8), two volunteer no wash controls had recovery concentrations <317 PFU/mL for Phi6. After decontamination changes (volunteers 9-26), an additional three volunteers had recovery concentrations <317 PFU/mL for Phi6. Although we cannot be certain why the no wash controls had limited recovery in some cases, we opted to keep all the collected data in the analysis to embrace the variability and imperfections in the scientific method. All MS2 no wash controls were >317 PFU/mL.

In these experiments, handwashing prior to the start of experiments was assumed to be sufficient to remove Phi6 and MS2 that may be present on volunteer's hands. Based on previous experiments in the same lab with a similar cohort, it is unlikely that volunteers would have contact with Phi6 and MS2 in the natural environment outside of the lab prior to the experiment.[1] To evaluate the effectiveness of decontamination between each handwashing method, decontamination tests were run prior to the beginning of the formal experiments. A >7 LRV was observed in viral concentration after decontamination, which was sufficient to not affect the results of subsequent handwashing methods, as should the measured quantity of remaining virus be carried over after decontamination, it would be inconsequential compared to the applied virus quantity prior to handwashing treatments.

Plaque assay controls. Negative plaque assay controls consisted of only plating soft agar and the host bacterium and required no plaques. If the plaque assay negative control had one or more plaques (occurred twice), it indicated contamination and all samples from the volunteer were assayed again. Data reported in this study come from the re-run assays (where all negative controls were negative). Positive assay controls consisted of plating the diluted phage stock used as seed in the experiments were plated as a positive control. Positive assay control concentrations were required to provide the expected number of PFUs based on the concentration of the stock solution concentration (10^{11} PFU/mL) for each virus. Positive assay controls performed as expected for all experiments. TSB used to recover the virus from hands, make dilutions, and propagate *E. coli* was tested as a negative control approximately 20 times in a separate set of experiments, and all controls were negative.

cDNA standards. For creation of cDNA standards, gel electrophoresis consisted of using a 1.5% agarose gel in 1xTAE buffer at 100 V for 60 min. A QIAquick Gel Extraction kit (Qiagen) was used to extract cDNA from the gel. Sample cDNA concentration was determined through a Qubit fluorometer dsDNA Assay kit (Life Technologies) and standards were diluted to 10^7 cp/4 μL, aliquoted, and stored at -80°C until use in RT-qPCR. To construct our standard curve, a range of 10-10^6 cp/4 μL standards were used.

[1] Anderson CE, Boehm AB., 2021. Transfer Rate of Enveloped and Nonenveloped Viruses between Fingerpads and Surfaces. *Applied and Environmental Microbiology* 87: e01215-21

Table S1: Handwashing Methods

#	Code	Full Name	Description
1	NDS	New Dry Supertowel	A brand-new Supertowel was used. It was not wetted. The volunteer rubbed all parts of their hands (palm, back of hand, fingers, and wrist) for 20 seconds.
2	NWS	New Wet Supertowel	A brand-new Supertowel was used. It was wetted by completely submerging the towel, then squeezing out approximately 125 mL of the water in the towel to leave the towel damp. The volunteer rubbed all parts of their hands (palm, back of hand, fingers, and wrist) for 20 seconds.
3	UDS	Used Dry Supertowel	A used Supertowel was used. It was not wetted. A used Supertowel was one that had been through the washing and drying process 10 times. Towels were hand washed for 10 minutes using 40 g powdered laundry detergent (Procter & Gamble, Cincinnati, OH, USA) and 10 L of water and then air dried. The volunteer rubbed all parts of their hands (palm, back of hand, fingers, and wrist) for 20 seconds.
4	UWS	Used Wet Supertowel	A used Supertowel was used. It was wetted by completely submerging the towel, then squeezing out approximately 125 mL of the water in the towel to leave the towel damp. A used Supertowel was one that had been through the washing and drying process 10 times. Towels were hand washed for 10 minutes using 40 g powdered laundry detergent and 10 L of water and then air dried. The volunteer rubbed all parts of their hands (palm, back of hand, fingers, and wrist) for 20 seconds.
5	NDiS	New Dirty Supertowel	A brand-new Supertowel was used. It was wetted by completely submerging the towel in dirty water, then squeezing out approximately 125 mL of the water in the towel to leave the towel damp. Dirty water was created by adding 5g of autoclaved soil (Gro Tec, Inc., Madison, GA, USA) to 1L of water. The volunteer will rub all parts of their hands (palm, back of hand, fingers, and wrist) for 20 seconds.
6	UDiS	Used Dirty Supertowel	A used Supertowel was used. It was wetted by completely submerging the towel in dirty water, then squeezing out approximately 125 mL of the water in the towel to leave the towel damp. Dirty water was created by adding 5g of autoclaved soil to 1L of water. A used Supertowel was one that has been through the washing and drying process 10 times. Towels were hand washed for 10 minutes using 40 g powdered laundry detergent and 10 L of water and then air dried. The volunteer rubbed all parts of their hands (palm, back of hand, fingers, and wrist) for 20 seconds.
7	SW20	Soap and Water, 20s	Soap (Colgate-Palmolive Company, New York, NY, USA) was applied to the hands and volunteers distributed the soap across all parts of their hands (palm, back of hand, fingers, and wrist). Volunteers then washed with soap and running water for 20 seconds. Volunteers were asked to dry their hands with a paper towel.
8	SW5	Soap and Water, 5s	Soap was applied to the hands and volunteers distributed the soap across all parts of their hands (palm, back of hand, fingers, and wrist). Volunteers then washed with soap and running water for 5 seconds. Volunteers were asked to dry their hands with a paper towel.

Table S1: Handwashing Methods (continued)

9	S20	Soapy Water, 20s	Volunteers washed all parts of their hands (palm, back of hand, fingers, and wrist) under a tap of soapy water for 20 seconds. Soapy water was created by adding 30 g of powdered laundry detergent (Procter & Gamble, Cincinnati, OH, USA) to 1.5 L water. Soapy water from the tank tap had a flow of approximately 0.08 L/s, while flow from the sink tap had a flow of approximately 0.09 L/s. Volunteers were asked to dry their hands with a paper towel.
10	W20	Water, 20s	Volunteers washed all parts of their hands (palm, back of hand, fingers, and wrist) in running water for 20 seconds. Volunteers were asked to dry their hands with a paper towel.
11	ABHS	Alcohol-Based Hand Sanitizer	Hand sanitizer (62% Ethyl alcohol, Target Corp., Minneapolis, MN, USA) was applied to the hands and volunteers distributed the sanitizer across all parts of their hands (palm, back of hand, fingers, and wrist) for 20 seconds. Hands then air dried.
12	WR	Wet Regular Towel	A brand-new regular towel was used. It was wetted by completely submerging the towel, then squeezing out approximately 125 mL of the water in the towel to leave the towel damp. The volunteer rubbed all parts of their hands (palm, back of hand, fingers, and wrist) for 20 seconds.
13	NW	No Wash Control	No wash occurred.

Table S2: Temperature, Humidity, and Handwashing Data

Volunteer #	Date	Humidity	Temperature	Hand	Order of Washing
1	27-May	42	20.8	Right	7,2,4,13,12,3,6,8,10,9,1,11,5
2	4-Jun	55	20.5	Left	10,8,5,6,9,1,4,12,11,13,3,2,7
3	8-Jun	39	21	Left	10,4,2,6,13,5,1,3,9,7,11,8,12
4	15-Jun	50	20.5	Right	12,11,5,7,1,2,4,13,9,8,6,10,3
5	23-Jun	56	20.5	Right	5,2,3,10,12,4,1,11,8,9,7,6,13
6	24-Jun	55	20.7	Right	3,12,4,1,10,6,5,7,11,2,9,13,8
7	28-Jun	59	20.8	Right	13,10,2,1,3,11,6,7,5,12,8,9,4
8	30-Jun	61	21.1	Right	11,12,10,9,13,1,7,4,5,2,8,3,6
9	5-Jul	62	20.6	Left	9,5,8,1,7,4,13,6,10,3,2,11,12
10	7-Jul	55	20.8	Left	5,10,7,9,11,8,3,12,2,4,6,13,1
11	27-Jul	64	20.9	Left	9,10,4,11,8,5,3,13,1,6,2,12,7
12	28-Jul	67	20.8	Right	10,4,11,6,8,9,2,13,3,7,1,5,12
13	30-Jul	67	21.2	Right	11,5,1,4,10,6,7,8,2,13,12,3,9
14	3-Aug	59	21	Right	9,6,8,10,5,2,11,1,7,4,13,12,3
15	4-Aug	60	21.1	Right	10,12,4,3,6,9,8,5,1,13,2,7,11
16	12-Aug	65	21.2	Left	1,6,12,5,9,4,3,13,11,2,7,10,8
17	13-Aug	65	21.1	Right	8,2,3,13,4,6,10,9,1,5,11,12,7
18	16-Aug	68	20.9	Right	2,4,13,11,10,8,3,7,1,5,9,6,12
19	24-Aug	60	21.1	Left	6,10,4,5,11,3,13,8,9,7,2,12,1
20	26-Aug	59	21.2	Left	1,11,8,10,5,4,13,2,6,3,12,9,7
21	27-Aug	57	20.1	Left	1,4,2,11,8,6,13,10,12,9,3,5,7
22	2-Sep	52	21.2	Left	5,7,2,10,8,6,11,1,12,13,4,9,3
23	6-Sep	59	21.2	Left	2,8,12,9,1,4,13,5,3,11,6,7,10
24	9-Sep	65	21.4	Left	8,11,1,3,13,2,7,4,9,12,5,10,6
25	14-Sep	54	20.7	Right	10,5,11,4,1,2,6,7,3,9,12,8,13
26	17-Sep	54	21.1	Right	6,11,10,7,13,3,5,2,9,12,1,4,8

Order of Washing Key

1 = New Dry Supertowel (NDS)	*8 = Soap and Water, 5s (SW5)*
2 = New Wet Supertowel (NWS)	*9 = Soapy Water, 20s (S20)*
3 = Used Dry Supertowel (UDS)	*10 = Water, 20s (W20)*
4 = Used Wet Supertowel (UWS)	*11 = ABHS (ABHS)*
5 = New Dirty Supertowel (NDiS)	*12 = Wet Regular Towel (WR)*
6 = Used Dirty Supertowel (UDiS)	*13 = No Wash Control (NW)*
7 = Soap and Water, 20s (SW20)	

Figure S1: Experimental Setup Photos

1. Application of the virus onto hands	2. Handwashing method example<br>a. Supertowel<br>b. Water only	3. TSB recovery
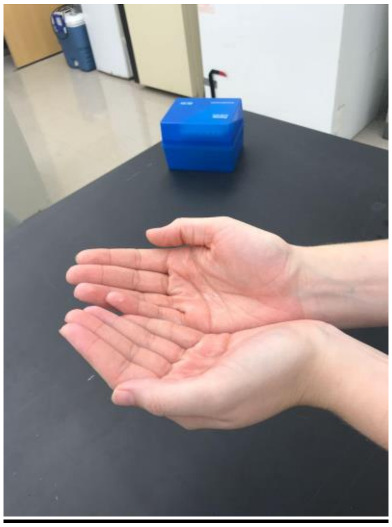	a.<br>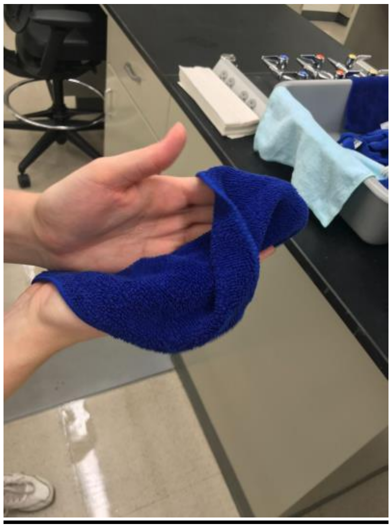	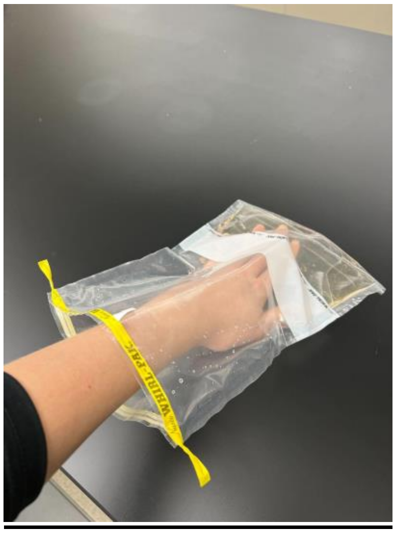
	b.<br>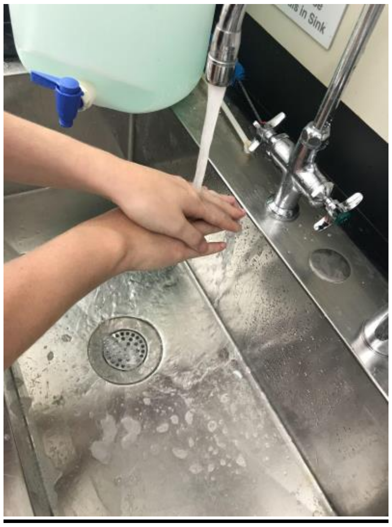	

Experimental Power Calculations

Sensitivity power analysis for this study was calculated using G*Power (Statistical power analyses using G*Power 3.1: Tests for correlation and regression analyses, version 3.1.9.6; University of Kiel, Germany).

Information about sample size is located in the Distribution of Data and Normality Tests section of the SM. The following variables were input into the program:

**Variable**	**Input**
Test family	t tests
Statistical test	Means: Wilcoxon signed-rank test (matched pairs)
Type of power analysis	Sensitivity: Compute the required effect size = given alpha, power, and sample size
Tail(s)	Two
Parent distribution	Normal
Alpha error probability	0.05
Power	0.80
Total sample size	52 (26 observations in each group)<br>10 (5 observations in each group)

The program was run two times, resulting in two different effect sizes, for each total sample size. The effect size *d* the sample population of 52 and 10 were able to detect are 0.41 and 1.03, respectively. These values can be compared to the conventional values proposed by Cohen (1969), where effect sizes were defined as 0.2 as small, 0.5 as medium, and 0.8 as large.

All significant p-values reported had effect sizes (Z statistic divided by square root of the sample size) greater than the minimum effect size the sample population was able to detect.

Table S3: RT-qPCR Targets

Amplicon	Forward Primer	Reverse Primer	Starting Position	Ending Position	Size (# bases)	Genome Fraction	Source
**MS2 DSMZ 13767 genome length 3569**							
MP6	CCTAAAGTGG CAACCCAGAC	AAAGATCGCG AGGAAGATCA	1530	1818	289	8.10%	Loeb et al. [31]
**Phi6 genome length ~13,380**							
M	CCTGAGGAAA CGGCTCAACT	CATAGCCAAC GAACTGCTGC	1307	1778	472	3.53%	Ye et al. [32]

Inhibition Testing

Inhibition was tested using 5 of the 25 samples analyzed for RT-qPCR. To choose the five samples, a list of random numbers was generated and the first five samples which represented the five handwashing methods tested were chosen. Samples were tested at 1:10 and 1:100 dilutions; 4 out of 5 samples were equivalent within a 95% confidence interval for both MP6 and M. The difference in mean sample concentration for the sample which was not equivalent was 0.05 log for MP6 and 0.26 log for M. Using this information, we concluded that the samples were not inhibited at the 1:10 dilution and proceeded with RT-qPCR quantification at a 1:10 dilution for all samples.

Figure S2: RT-qPCR Inhibition Testing

Figure S2 shows the concentration*dilution of RT-qPCR samples versus the dilution tested for each of the five samples tested. The bars on the points represent the 95% confidence intervals.

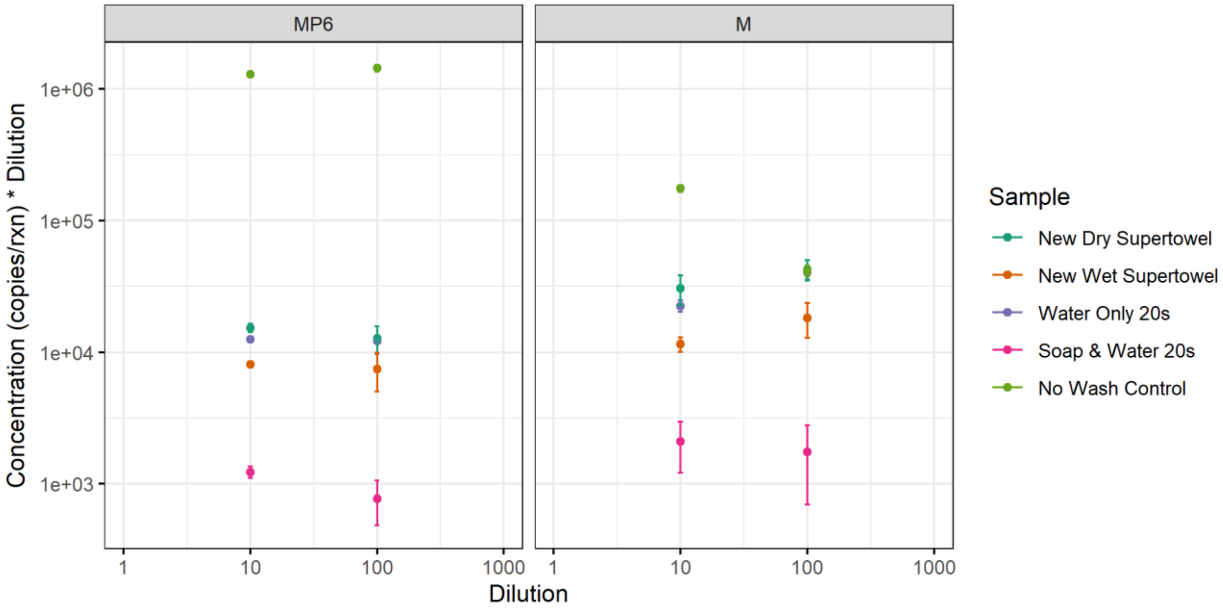

Environmental Microbiology Minimum Information (EMMI) Checklist

Study Description

Study: Handwashing Study
Date: January 2022
Completed by: C. Anderson

Environmental Sampling	Sample Treatment	Sample Reduction	Nucleic Acid Extraction	Reverse Transcription	PCR Detection	Analysis
N/A	☐ Performed<br>N/A	☐ Performed<br>N/A	QIAamp Viral RNA Mini kit used for Phi6 and MS2.	■ Performed<br>One-step used for Phi6 and MS2.	■ qPCR ☐ dPCR<br>One-step RT-qPCR was used for Phi6 and MS2. Samples were quanitfied in technical duplicates.	Thresholds were manually set on the StepOnePlus™ Real-Time PCR System. Standard curves were averaged for all plates. Technical duplicates were also averaged.

Control Checklist

	Environmental Sampling	Sample Treatment	Sample Reduction	Nucleic Acid Extraction	Reverse Transcription	PCR Detection
Step performed	☐	☐	☐	■	■	■
**Negative Controls**						
Step has control info	☐	☐	☐	■	☐	■
# control replicates	0	0	0	2	0	2
Control result reported	☐	☐	☐	■	☐	■
Data handling reported	☐	☐	☐	■	☐	■
**Positive Controls**						
Control introduced	☐	☐	☐	■	☐	■
Internal/External	N/A	N/A	N/A	Internal	N/A	Internal
Independent/Parallel	N/A	N/A	N/A	Independent	N/A	Independent
Step has control info	☐	☐	☐	■	☐	■
# control replicates	0	0	0	2	0	2
Control result reported	☐	☐	☐	■	☐	■
Data Handling reported	☐	☐	☐	■	☐	■

Process Checklist

Environmental Sampling

- ☐ Sampling Procedure
- ☐ Number of samples
- ☐ Sample amount, mean, range
- ☐ Sampling locations, dates, times

Sample Treatment

- ☐ Performed
- ☐ Treatment procedure
- ☐ Reagents

Sample Reduction

- ☐ Performed
- ☐ Reduction procedure
- ☐ Reagents
- ☐ Concentration Factor

Nucleic Acid Extraction

- ■ Extraction procedure
- ■ Amount extracted, amount obtained
- ■ Extract storage conditions

qPCR or dPCR

- ■ Target gene name, amplicon length
- ■ Thermocycling temperatures and times
- ■ Master mix: composition, vendors, concentrations
- ■ Additives: vendors, concentrations
- ■ Template amount added, pre-treatment (if any)
- ■ Primers: sequences, concentrations, vendors, references
- ■ Amplicon confirmation method (probe, melt curve, etc)
- ☐ Probe sequence, concentration, vendor, reference
- ■ Instrumentation
- ■ Equivalent volume of sample analyzed by PCR
- ■ Inhibition assessment procedure
- ■ Inhibition control description (if used)
- ■ Number samples tested and found inhibited

Reverse Transcription

- ■ Performed
- ■ One or two step
- ☐ cDNA storage conditions (if two step)
- ■ Reaction temperatures and times
- ■ Reaction reagents and concentrations
- ■ Priming method
- ■ Reaction volume, added template amount
- ■ Inhibition assessment procedure
- ■ Inhibition control description (if used)
- ■ Number samples tested and found inhibited

Analysis – dPCR

- ☐ Threshold settings
- ☐ Technical replicates, number, well merging
- ☐ Partitions measured, number, mean, variance
- ☐ Partition volume
- ☐ Target copies per partition, mean, variance
- ☐ Program used for dPCR analysis
- ☐ Explanation of control results, example plots

Analysis – qPCR

- ■ Method for handling failed negative controls
- ■ Technical replicates, number, calculations
- ■ Calibration standards: description and source
- ■ Method of quantifying standards
- ■ Calibration curve slope
- ■ Calibration curve R2
- ■ Lowest standard measured or 95% LOD
- ■ Cq value determination method

MS2 and Phi6 Handwashing Heat Maps

Figures below show the results of statistical tests between handwashing methods. Significant p-values have no pattern, while non-significant p-values are striped. Effect sizes are filled with different colors, with white being the lowest effect size (smallest difference measured) and the red being the largest effect size (largest difference measured). Wilcoxon effect sizes range from 0-1, with delimiters 0.2 as small, 0.5 as medium, and 0.8 as large. Our study was powered for effect sizes of 0.4.

Figure S3: MS2 Heat Map

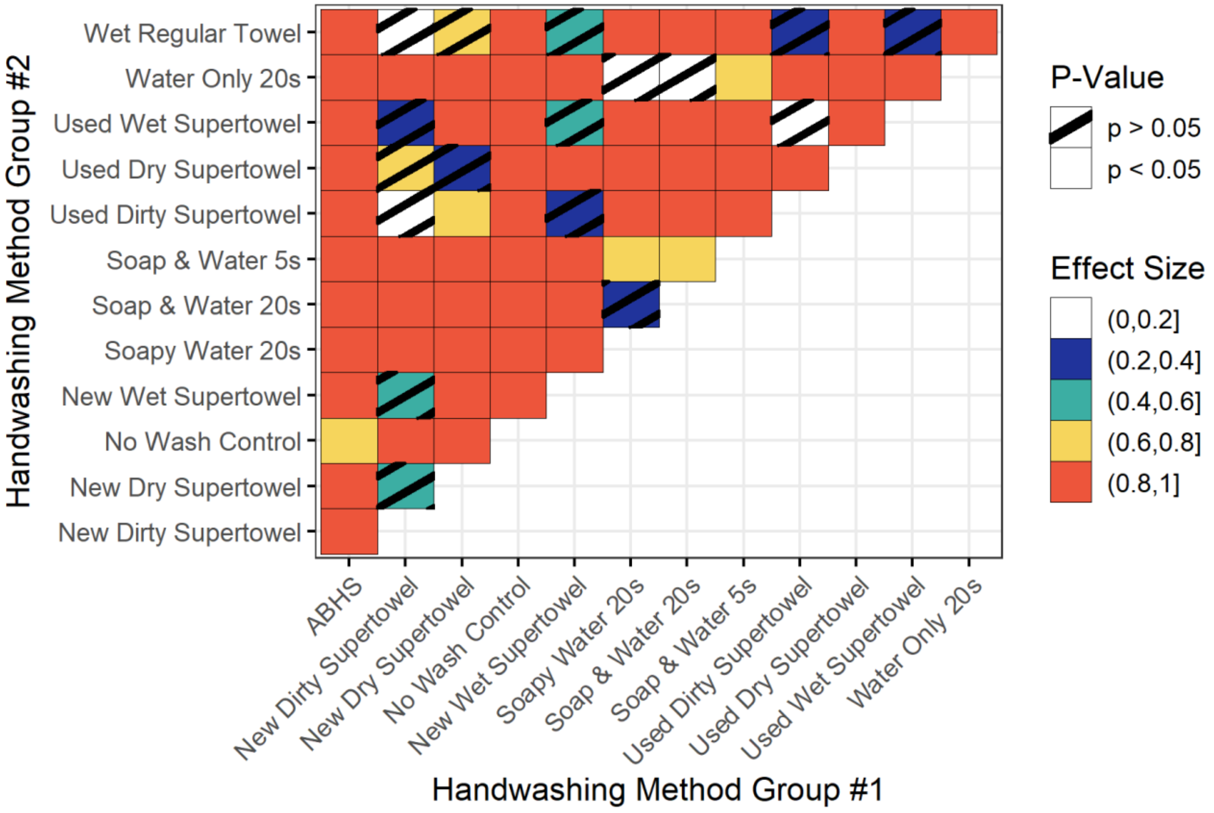

Figure S4: Phi6 Heat Map

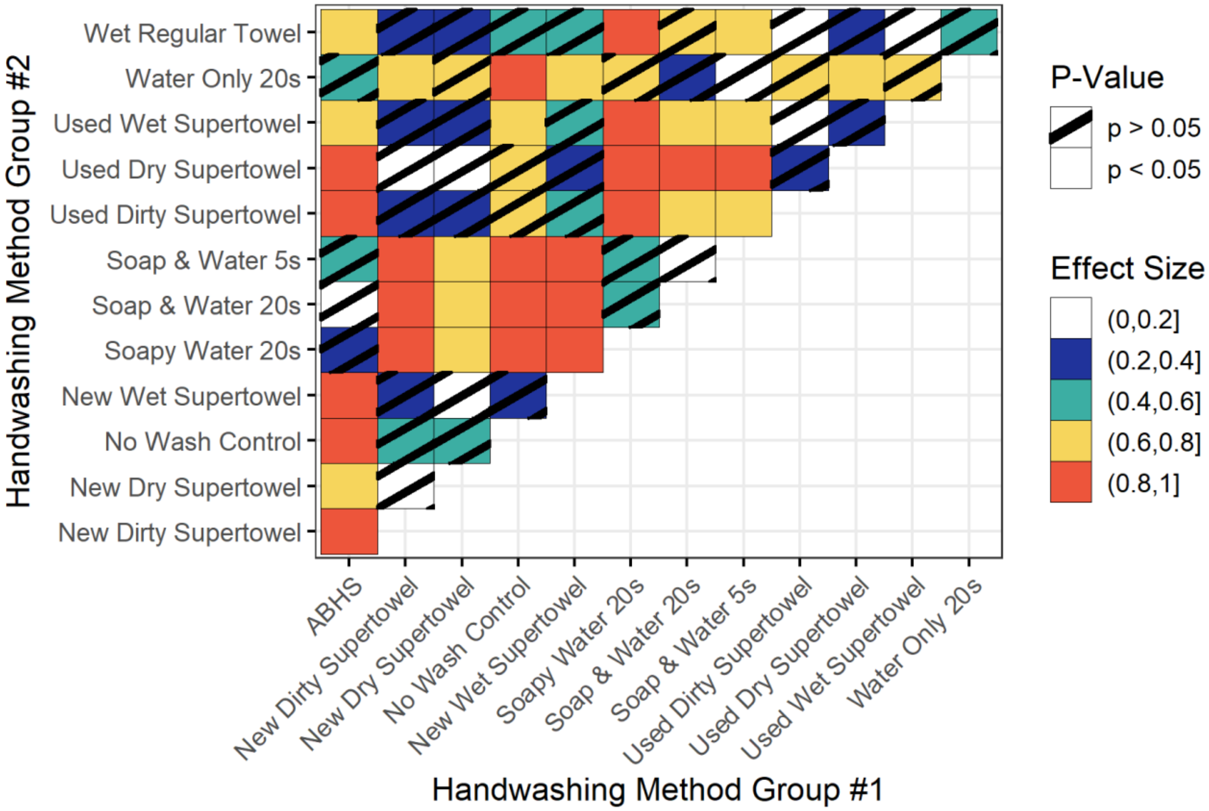

Figure S5: Standard Curves and Master Equations

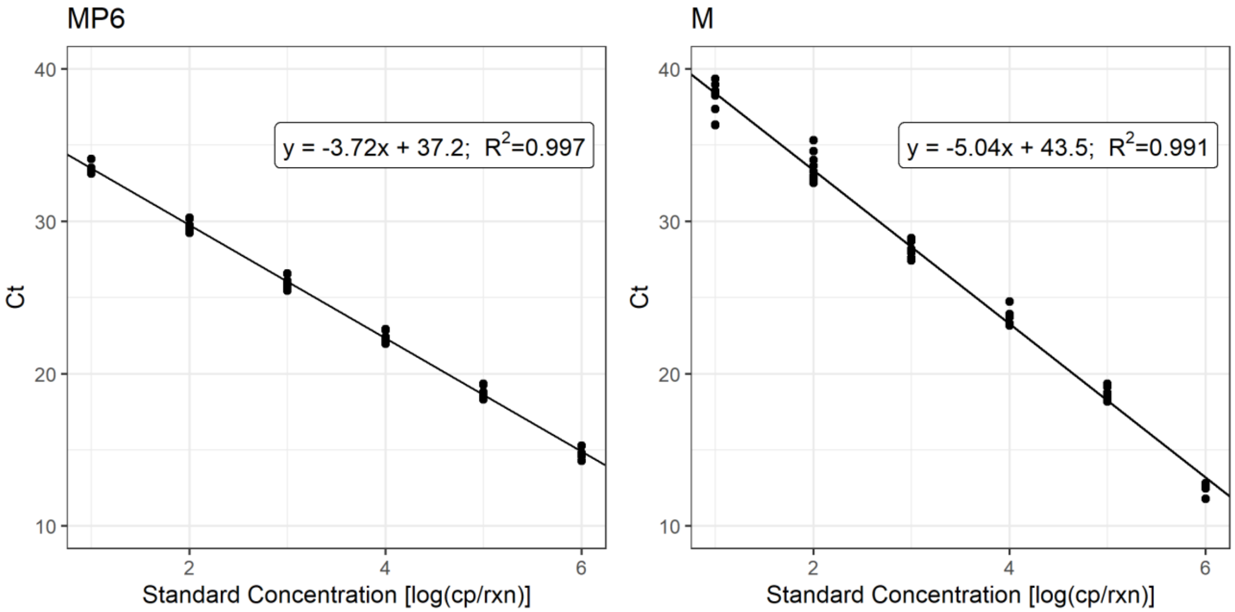

Distribution of Data and Normality Tests

To assess if the data were parametric, the distribution of data were plotted, Q-Q plots were made, Shapiro-Wilk tests were run, and equality of variances Bartlett tests were run for each subset of data tested. For each subset, if the distribution was not normal (Shapiro-Wilk tests P-value < 0.05), the Q-Q plot was not linear, or the variances were not homogenous (Bartlett test P-value < 0.05), the data were deemed non-parametric. Because a majority of the data were non-parametric, non-parametric tests were used throughout the data analysis.

All plaque assay data distribution plots, Q-Q plots, and normality statistical tests for MS2 and Phi6 are shown in Figure S6 and S7, respectively. A summary of the normality tests and Q-Q plots for RT-qPCR and the subset of volunteers from plaque assays used in RT-qPCR analysis are in Table S4.

Figure S6: MS2 Plaque Assay Normality Tests (All Volunteers)

The p-value from the Bartlett test (P=0.99) indicates homogeneity of variance. Shapiro-Wilk test P-values are shown in figure labels. Sample size equals 26 for each group.

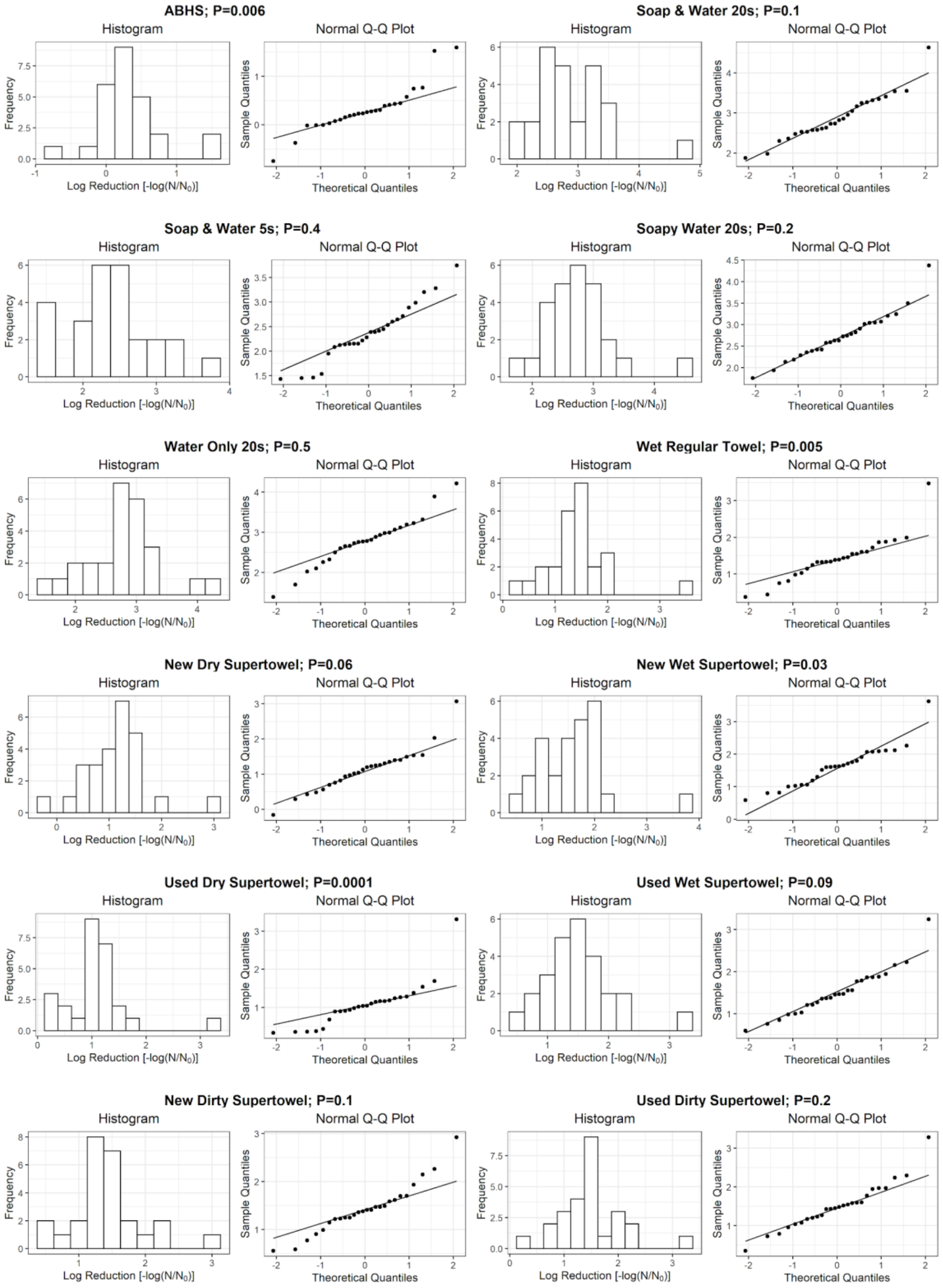

Figure S7: Phi6 Plaque Assay Normality Tests (All Volunteers)

The p-value from the Bartlett test (P=0.10) indicates homogeneity of variance. Shapiro-Wilk test P-values are shown in figure labels. Sample size equals 26 for each group.

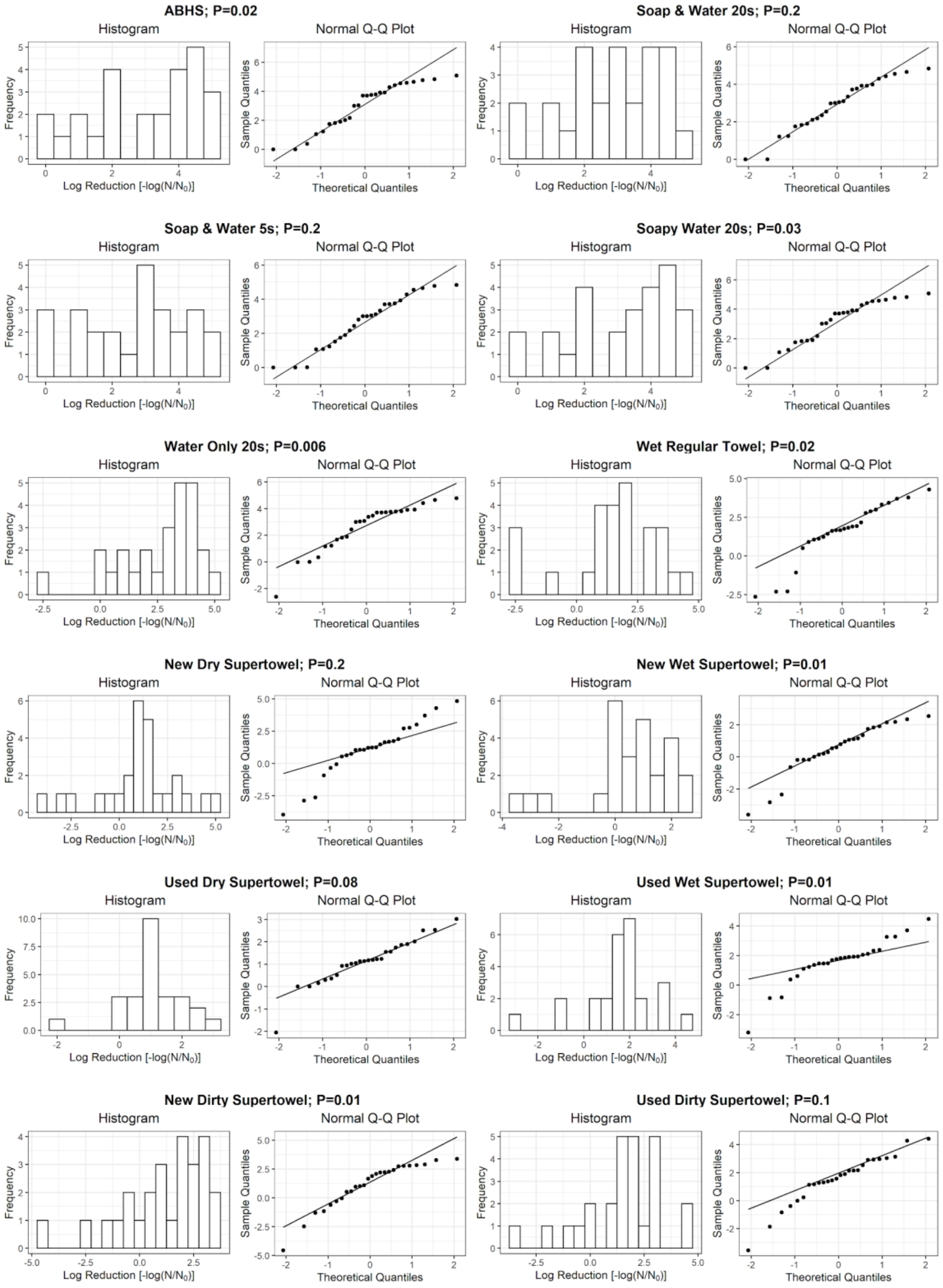

Table S4: Normality test results for data subsets

Assay	Target (if applicable)	Handwashing Method	n	Bartlett Test	Shapiro-Wilk Test	Points appear linear in Q-Q plot?
RT-qPCR	MP6	Soap & Water 20s	5	0.69	0.4	No
		Water Only 20s	5		0.1	No
		New Dry Supertowel	5		0.2	No
		New Wet Supertowel	5		0.4	No
	M	Soap & Water 20s	5	0.73	0.03	Yes
		Water Only 20s	5		0.7	No
		New Dry Supertowel	5		0.3	Yes
		New Wet Supertowel	5		0.2	No
MS2 Plaque Assay (5 volunteer RT-qPCR subset)	N/A	Soap & Water 20s	5	0.66	0.6	Yes
		Water Only 20s	5		0.08	No
		New Dry Supertowel	5		0.004	No
		New Wet Supertowel	5		0.8	Yes
Phi6 Plaque Assay (5 volunteer RT-qPCR subset)	N/A	Soap & Water 20s	5	0.76	0.2	Yes
		Water Only 20s	5		0.6	Yes
		New Dry Supertowel	5		0.3	Yes
		New Wet Supertowel	5		0.7	Yes
